# DNA Damage and Parkinson’s Disease

**DOI:** 10.3390/ijms25084187

**Published:** 2024-04-10

**Authors:** Gerd P. Pfeifer

**Affiliations:** Department of Epigenetics, Van Andel Institute, Grand Rapids, MI 49503, USA; gerd.pfeifer@vai.org

**Keywords:** Parkinson’s disease, DNA damage, mitochondria, DNA repair, transcription, mutations

## Abstract

The etiology underlying most sporadic Parkinson’s’ disease (PD) cases is unknown. Environmental exposures have been suggested as putative causes of the disease. In cell models and in animal studies, certain chemicals can destroy dopaminergic neurons. However, the mechanisms of how these chemicals cause the death of neurons is not understood. Several of these agents are mitochondrial toxins that inhibit the mitochondrial complex I of the electron transport chain. Familial PD genes also encode proteins with important functions in mitochondria. Mitochondrial dysfunction of the respiratory chain, in combination with the presence of redox active dopamine molecules in these cells, will lead to the accumulation of reactive oxygen species (ROS) in dopaminergic neurons. Here, I propose a mechanism regarding how ROS may lead to cell killing with a specificity for neurons. One rarely considered hypothesis is that ROS produced by defective mitochondria will lead to the formation of oxidative DNA damage in nuclear DNA. Many genes that encode proteins with neuron-specific functions are extraordinary long, ranging in size from several hundred kilobases to well over a megabase. It is predictable that such long genes will contain large numbers of damaged DNA bases, for example in the form of 8-oxoguanine (8-oxoG), which is a major DNA damage type produced by ROS. These DNA lesions will slow down or stall the progression of RNA polymerase II, which is a term referred to as transcription stress. Furthermore, ROS-induced DNA damage may cause mutations, even in postmitotic cells such as neurons. I propose that the impaired transcription and mutagenesis of long, neuron-specific genes will lead to a loss of neuronal integrity, eventually leading to the death of these cells during a human lifetime.

## 1. Introduction

Parkinson’s disease (PD) affects about one million people in the United States and ten million worldwide. The number of Parkinson’s disease cases has more than doubled over the past 30 years. There is limited information about the causation of this increase. The disease is characterized by motor symptoms and non-motor symptoms. There is a severe loss of dopaminergic neurons in the substantia nigra pars compacta and the ventral midbrain, and this loss of neurons is responsible for the motor symptoms. Only 5 to 10% of PD cases can be linked to autosomal inherited gene defects (familial PD) in about a dozen or so different genes [1]. The origin of most idiopathic PD cases is unknown, although extensive genome-wide association studies (GWAS) have revealed combinations of variants that may increase susceptibility to the disease [2]. Aging is a prominent risk factor, because non-inherited disease rarely occurs in individuals below 60 years of age. For these reasons, we need to contemplate what type of general or neuron-specific decay mechanisms become more prominent in aged individuals. The hallmarks of aging include epigenetic alterations, loss of proteostasis, disabled autophagy, deregulated nutrient sensing, cellular senescence, altered intercellular communication, chronic inflammation, dysbiosis, and genomic instability [3]. While there is good evidence for a role of dysfunctional proteostasis, autophagy, and inflammation in PD [4,5], the causative involvement of epigenetic alterations and genomic instability in the disease are less clear and are just at the beginning of being understood. The role of epigenetic alterations in PD has been reviewed elsewhere [6,7]. Here, I will focus on the potential role of genomic instability in Parkinson’s disease.

## 2. DNA Damage and Parkinson’s Disease—The Intriguing Case of Trichloroethylene

In addition to aging and the multigene predisposition effects as recorded by GWAS, environmental exposures have been suggested as putative causes of PD [8,9]. However, the evidence for that connection is not fully established, which is mainly because of the difficulty in quantifying specific single exposures or mixed exposures over a lifetime in sufficiently powered population cohorts. There are a few notable exceptions, where a strong link between an exposure and PD has been made. A recent study analyzed the health records of over 80,000 veterans stationed at Camp Lejeune in North Carolina who were exposed to the chemical trichloroethylene (TCE) due to a contamination of the water supply at the military base [10]. The water consumed by the military personnel contained levels of the TCE chemical that were more than 70 times higher than the level allowed by the U.S. Environmental Protection Agency. The authors compared the data from the North Carolina cohort with a similar number of veterans stationed at a non-contaminated base in California. They reported that the TCE-exposed population had a 70% increased risk of PD, which was highly significant given the large number of individuals analyzed [10,11]. TCE has been widely used as a degreasing and cleaning agent and is found in numerous other consumer products [12]. This volatile substance is present in indoor and outdoor air and contaminates groundwater in many parts of the world. TCE mass production started at the beginning of the 20th century, with millions of tons of this chemical have been produced to date. Such epidemiological studies as this one reported recently, in addition to the smaller cohorts examined in the past, are highlighting the fact that further research into the contribution of this chemical to the etiology of PD is required.

TCE is a liquid that easily crosses biological membranes and penetrates the blood–brain barrier [13]. In cell models and in animal experiments, TCE can destroy dopaminergic neurons [13,14,15,16]. TCE is a mitochondrial toxin that inhibits the mitochondrial complex I, and three of its metabolites have also been linked to mitochondrial dysfunction [16,17,18]. These compounds have all been shown to cause a loss of dopaminergic neurons from the nigrostriatal tract in rodents.

## 3. Mitochondrial Dysfunction, Mitochondrial Toxins, and Parkinson’s Disease

In addition to TCE, a number of other chemicals are known to inhibit mitochondrial complex I function and are toxic to dopamine neurons, including rotenone, paraquat, 1-methyl-4-phenyl-1,2,3,6-tetrahydropyridine (MPTP) and 6-hydroxydopamine (6-OHDA) (Table 1) [19,20]. Mitochondrial dysfunction leads to a reduction in oxidative phosphorylation and ATP production associated with the overproduction of reactive oxygen species (ROS) by enhanced electron leakage from the defective electron transport chains [21,22].

Rotenone and paraquat have been used as pesticides in the agricultural industry. Rotenone, an isoflavone compound, is found in some plants of the legume family and has historically been used for killing fish and insects. This substance is now banned in the US and most other countries for use in agriculture but still is being applied for killing invasive species of fish. By inhibiting the electron transport chain in mitochondria, rotenone leads to a backup of electrons and reduction in cellular oxygen, which creates oxygen radicals and other reactive oxygen species. Rotenone is only moderately toxic to mammals including humans because it is not easily absorbed. However, the injection of rotenone into rats produces Parkinson-like symptoms [23]. A study of farm workers who had used rotenone-containing pesticides indicated an increased risk (odds ratio = 2.5) of developing PD compared to controls [24]. In cultured neurons, concentrations of rotenone in the nanomolar range will lead to cell killing [25].

Paraquat is one of the most commonly used herbicides in the agricultural industry, still today, even though this substance has been linked to PD since 1987 [26]. A meta-analysis of 13 case-control studies with 3231 PD patients and 4901 controls revealed an association between PD and paraquat exposure at an odds ratio of 1.64 [27]. A more recent study considering residential and workplace proximity to commercial agricultural paraquat application sites in California confirmed this association with an odds ratio of about 2 when over 800 PD patients or about 800 controls were analyzed [28]. Like rotenone, paraquat is a mitochondrial toxin with weak complex I inhibiting activity, although it may have other toxic effects, and it causes the death of dopaminergic neurons in vitro and in animal models [29]. Paraquat is a redox-active compound and may produce ROS during its own redox cycling. This chemical induces senescence and a pro-inflammatory state in vitro and in vivo [30]. Low-dose paraquat animal models can recapitulate many features of the human disease including alpha-synuclein pathology [31].

Initially discovered after the self-administration of an illicit drug, N-methyl-4-phenyl-1,2,3,6-tetrahydropyridine (MPTP) (Table 1) is a potent agent to induce Parkinsonian-like syndromes in humans and in animal models [32,33,34]. MTPT is converted in the brain to its active metabolite, the MPP+ ion. Various MPTP animal models are now widely used in PD research. MPTP exposure leads to the degeneration of dopaminergic neurons in vivo and in vitro. This effect is believed to be a consequence of the inhibition of mitochondrial complex I by this compound.

**Figure 1:**
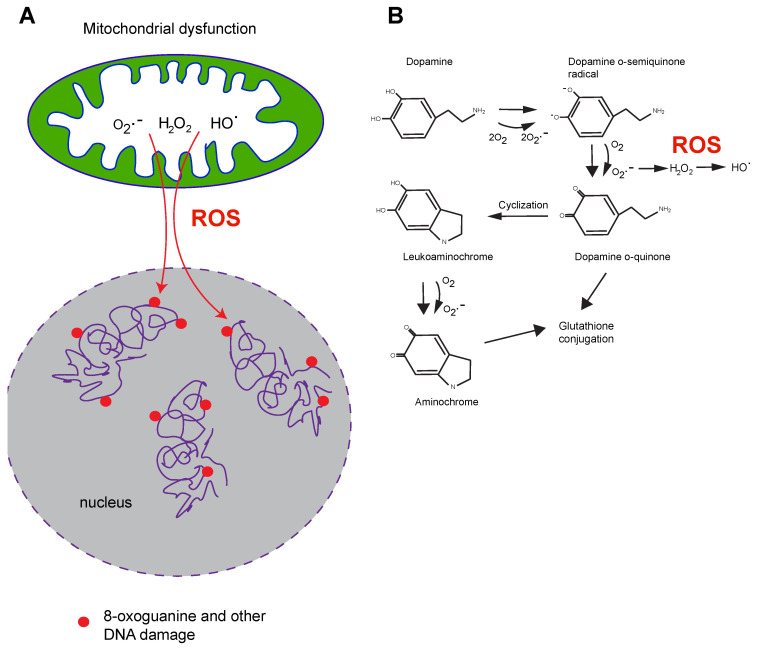
Production of reactive oxygen species (ROS) in dysfunctional mitochondria and by dopamine redox cycling. (**A**). ROS produced in dysfunctional mitochondria can diffuse into the nucleus to cause DNA damage. (**B**). Dopamine oxidation generates reactive oxygen species (ROS).

6-hydroxydopamine is a synthetic compound that has been used in animal models of PD in which it causes the loss of dopaminergic neurons. Its exact mechanism of action is unclear, but it has been linked to the production of oxidative stress and to mitochondrial dysfunction [35,36,37]. Like dopamine itself (Figure 1), several of the chemicals that induce PD-like symptoms are redox-cycling compounds that through their oxidation–reduction cycles can produce reactive oxygen species. This fact leads to a speculation that the presence of these types of chemicals in dopaminergic neurons may overwhelm the antioxidant defense systems of these cells, leading to macromolecular damage including damage to DNA.

From these studies, it has become clear that the dysregulation of mitochondrial homeostasis is an important process that occurs during pathogenesis, leading to neuronal loss in PD [38,39]. Mitochondrial dysfunction of the respiratory chain leads to the accumulation of ROS, such as hydrogen peroxide, superoxide anion and peroxyl radicals (Figure 1). Hydrogen peroxide and superoxide anion can be subsequently converted to the very reactive hydroxyl radical in the presence of iron via the Haber–Weiss and Fenton reactions and can damage nucleobases when this reaction occurs in proximity to DNA. A role of iron in the pathogenesis of PD has been discussed previously [40].

As for environmental exposures, heavy metals, such as iron, mercury, manganese, copper, and lead, have all been linked to PD [41]. These metals have the potential to disrupt redox homeostasis of the cell, can generate ROS, and may diminish antioxidant defense systems in dopaminergic neurons.

## 4. Defects of Mitochondrial Pathways in Familial and Sporadic PD

Several chemical agents known to induce Parkinson’s-like syndrome have in common that they cause mitochondrial dysfunction through inhibiting the electron transport chain by affecting mitochondrial complex I. However, the exposure of human populations to such chemicals is still relatively uncommon, except perhaps in the case of TCE, which is a ubiquitous environmental pollutant. This brings us to a discussion of how mitochondrial function may be defective in genetically inherited, early onset PD and in sporadic disease as a function of aging.

Familial PD-associated genes are often involved in a limited set of defined biological pathways. These pathways include lysosome function, autophagy, membrane trafficking and endocytosis, and the immune response. Another prominent pathway is mitochondrial function. For example, the protein PARKIN, an E3 ubiquitin ligase, and PINK1, a mitochondrial kinase, are clearly in the same biochemical pathway to support mitochondrial quality control [42]. Inherited mutations that lead to autosomal recessive PD have been found in the genes *PINK1* (*PARK6*), *PARK2* (*PARKIN*), *PARK7* (*DJ-1*), *CHCHD2*, *PARK13* (*HTRA2*), *PARK14* (*PLA2G6*), *PARK15* (*FBOX7*), and *VPS13C*, which all have functional roles in mitochondria [43] (Table 2). For late-onset PD, 14 mitochondrial function-associated genes have been identified in GWAS data sets [44]. Interestingly, one gene linked to familial PD is *DJ-1* (*PARK7*), which is a gene that encodes a protein with ROS-scavenging properties [45].

It needs to be discussed in this context that there is also contrarian evidence that argues against a role of complex I inhibition in PD. For example, a loss of complex I activity by deletion of the *Ndufs4* gene, encoding an accessory subunit of the mitochondrial membrane respiratory chain NADH dehydrogenase, did not cause dopaminergic neuron death in mice [56]. A recent review summarized the difficulties in assessing complex I levels and enzymatic activities in postmortem PD brain tissue and in peripheral tissues [57]. The authors emphasize that patients with rare inherited mutations in complex I subunits do not generally develop parkinsonian syndromes, but most of those patients do not live long enough to develop PD.

As with many cellular processes, mitochondrial function declines with aging. This functional decline can occur at the level of mutation accumulation with age, which is a phenomenon that is found in all tissues [58,59]. Mitochondrial DNA may be particularly vulnerable to the acquisition of mutations because of its rapid replication cycles, inefficient repair of mitochondrial DNA, and generation of large amounts of reactive oxygen species in these organelles. The mutations may affect the synthesis or function of mitochondrially encoded proteins. The mitochondrial genome encodes 13 genes which encode essential subunits of the oxidative phosphorylation (OXPHOS) enzymes. Because each cell contains thousands of copies of the mitochondrial genome, extensive replication over the lifespan of an individual will generate a mix of wild-type and mutant mitochondrial DNA molecules (heteroplasmy). These events include point mutations or deletions. However, it has been extremely challenging to draw reliable conclusions regarding whether somatic mtDNA deletions and point mutations are more prevalent in PD than in normal controls. These studies have often produced conflicting results, which is perhaps due to technical problems in analyzing mutations or because of underpowered study designs [60].

Mitochondrial dysfunction is clearly increased during normal aging [61]. Reduced proteostasis during aging may affect the stability and function of components of the electron transport chain. Although not all mitochondria in a cell may be compromised by these processes, a substantial fraction will be affected. As a result of this loss of mitochondrial functionality of the respiratory chain complexes during aging, more ROS will be produced, resulting in further damage to these organelles. Indeed, patients with sporadic PD have reduced complex I activity in different brain regions [62].

## 5. DNA Damage in PD

One major and appreciated mechanism of mitochondrial dysfunction in PD is the fact that the declining function of mitochondria will lead to bioenergetic defects in the form of an energy crisis, for example the reduced formation of ATP, which in turn may be detrimental for the viability of dopaminergic neurons.

However, here, I will discuss other possible outcomes and will focus on ROS-induced DNA damage. If mitochondrial dysfunction is a common, though perhaps not ubiquitous feature in familial and sporadic, age-associated PD, one can hypothesize that increased levels of ROS, which are a by-product in dysfunctional mitochondria, could damage DNA and cause genetic instability. Simply based on physical proximity, one would expect that mitochondrial DNA would be the first target for such damage to occur. Whether mitochondrial DNA damage would have an immediate impact on the disease is questionable. This damage may impede the transcription of mitochondrial genes or may cause mutations in replicating mitochondrial DNA. Long-term, however, these mitochondrial mutations are expected to further exacerbate mitochondrial dysfunction.

Furthermore, one other plausible outcome is damage to nuclear DNA. Some of the reactive oxygen molecules produced in dysfunctional mitochondria can be long-lived and can diffuse into the nucleus: for example, hydrogen peroxide. In addition to small oxygen-based molecules, ROS may cause membrane lipid peroxidation, leading to the formation of electrophilic aldehydes derived from unsaturated fatty acids. Brain tissue is rich in polyunsaturated fatty acid as a normal component of biological membranes. For example, 4-hydroxynonenal [63] is produced from lipids that contain polyunsaturated omega-6 fatty acids such as arachidonic acid and linoleic acid. These reactive aldehydes have a longer half-life than hydrogen peroxide or superoxide anion. Reactive oxygen species and lipid peroxidation products can promote the formation of DNA damage not only in mitochondrial DNA but also in nuclear DNA.

**Figure 2:**
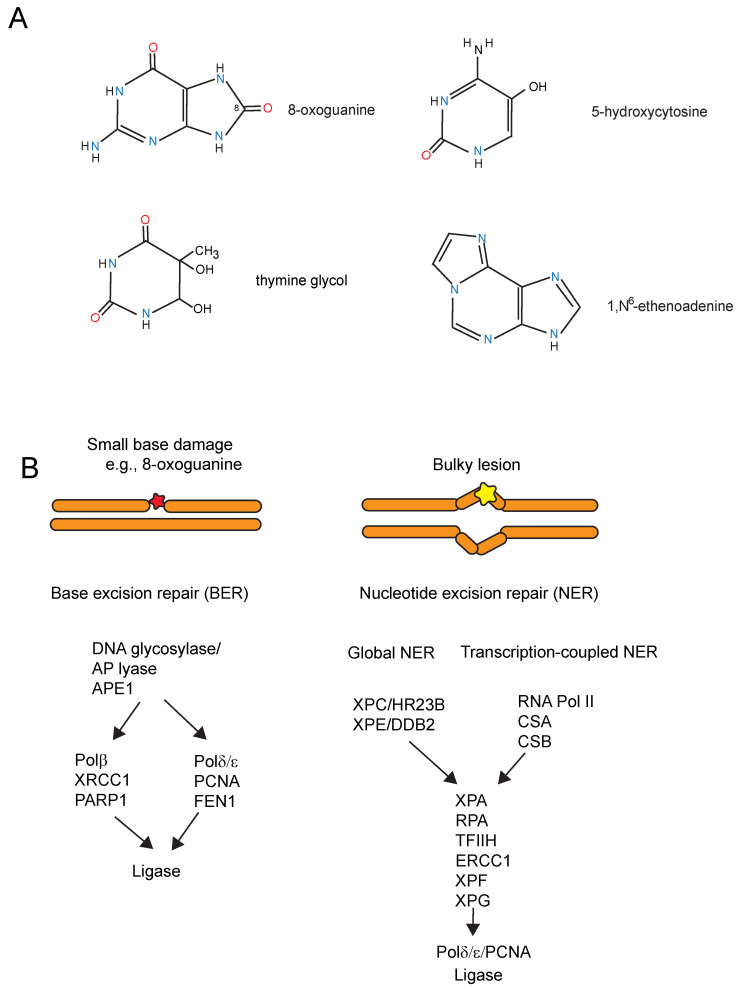
Major oxidative DNA damage products produced by ROS and DNA repair mechanisms. (**A**). Damaged DNA bases induced by ROS. (**B**). DNA repair mechanisms. Base excision repair (BER) is shown on the left. This pathway exists as two types of mechanisms, short-patch and long-patch BER that require different proteins. Nucleotide excision repair (NER) is shown on the right. This pathway is subdivided into global NER and transcription-coupled NER, which operates in transcribed genes. Key protein factors involved in the different repair mechanisms are shown.

ROS produce modified DNA bases and DNA strand breaks. The strand breaks will be mostly single-strand breaks, arising either directly because of damage to the sugar-phosphate backbone of DNA or indirectly occurring as intermediates of DNA repair processes. Occasionally produced DNA double-strand breaks, although rare, could lead to genome rearrangements such as translocations, deletions, insertions, or amplifications. DNA base damage induced by oxidative stress includes 8-oxoguanine (8-oxoG) as a prominent reaction product but also other modifications such as 5-hydroxycytosine, thymine glycol, or oxidized adenines (Figure 2A). The reactive aldehydes derived from lipid peroxidation can react with exocyclic amino groups of DNA bases to form, for example, DNA etheno-adducts such as 1,*N*^2^-etheno-guanine, *N*^2^,3-etheno-guanine, 1,*N*^6^-etheno-adenine (Figure 2A), and 3,*N*^4^-etheno-cytosine. All these modified bases are subject to DNA repair either by base excision repair or by nucleotide excision repair, depending mostly on the size of the base modification (Figure 2B). These DNA lesions have the potential to be mutagenic or interfere with transcription processes.

The levels of 8-oxoG have been shown to be specifically elevated in the substantia nigra of PD patients [64]. It should be mentioned in this context that the precise measurement of oxidized DNA bases in tissues has remained a technical challenge for several decades. It is difficult to measure these lesions when they occur at low frequencies when at the same time one needs to be able to avoid the background inherent to most DNA isolation methods. There is clearly a knowledge gap in assessing and understanding the extent of oxidative DNA damage in neuronal cells of the substantia nigra.

More sensitive technology, based on genome sequencing [65], are now available to undertake these difficult tasks, but they have not yet been applied in the PD field or in other research related to neurodegeneration. Importantly, dopaminergic neurons appear to be particularly vulnerable to oxidative stress [66,67]. These neurons not only require intensive mitochondrial respiration for proper function but also have limited inherent antioxidant capacity. This is particularly pertinent when we are also considering the reactive nature of the dopamine molecule (Figure 1) that is present in these neurons, making them further vulnerable to oxidative damage [66]. It is tempting to speculate that the selective vulnerability of dopaminergic neurons is related to these properties of the cells.

It has long been assumed that most mutations in human tissues are the product of processes linked to DNA replication except perhaps for mutagenesis linked to the hydrolytic or enzymatic deamination of DNA bases, i.e., the conversion of cytosine to uracil. The replication events would represent errors of the DNA polymerase or proofreading machineries, or they could be caused by the misincorporation of the wrong DNA bases when polymerases copy a DNA template that contains a base lesion. However, this assumption may be incorrect. Intriguingly, recent data suggest that nondividing (post-mitotic) cells such as neurons also accumulate mutations with the increasing age of the individual [58]. In fact, the rate of mutation accumulation with age was similar in dividing and nondividing tissues. The mechanisms of mutation accumulation in aging neurons are not clear at present, but they could perhaps be linked to errors introduced during the DNA repair of certain lesions. Using advanced sequencing technologies with low error rates, mutations can now be measured in neurons, even in single cells. Interestingly, an accumulation of C to A (G to T) mutations has been shown in Alzheimer’s disease brains [68]. Such mutations are theoretically the result of the mutagenic bypass of 8-oxoguanine lesions when an adenine becomes incorporated opposite to 8-oxoG. No such mutation studies have been reported yet for Parkinson’s disease brain.

## 6. Inflammation and PD

**Figure 3:**
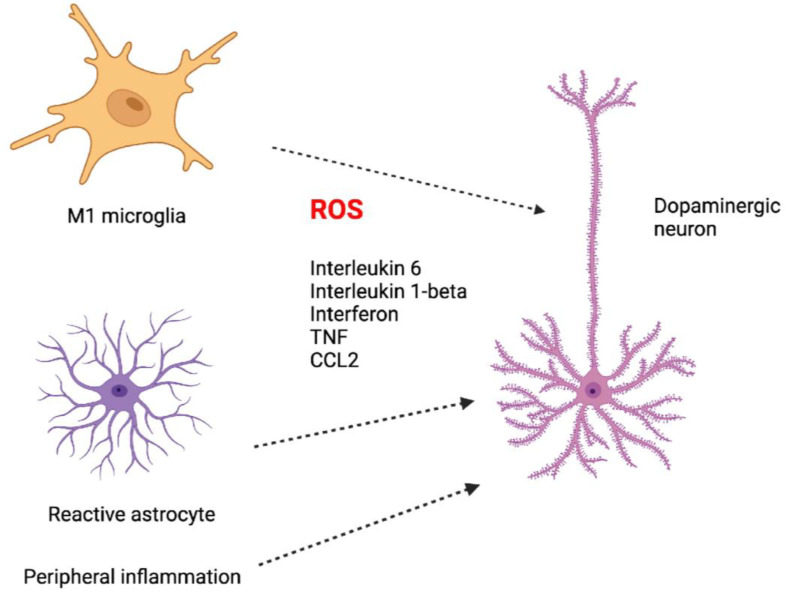
Production of ROS and cytokines by inflammatory processes in the brain. Created with Biorender.com. Accessed 18 March 2024.

In addition to the environmental exposures discussed above, inflammation is increasingly linked to neurodegeneration [69,70]. Microglia are resident brain cells that respond to injury or toxic agents that induce their proliferation and activation to release immune regulators, growth factors, and neurotoxic reactive chemicals (Figure 3). Chronic inflammation produces additional oxidative stress in the form of ROS released from microglia. There has been a debate as to whether neuroinflammation is a consequence of PD or whether it may be a primary cause of neurodegeneration. Alpha-synuclein accumulating in microglia induced a strong reactive state of these cells with an excessive production of various ROS and pro-inflammatory cytokines, leading to the cell death of neighboring neurons [71]. In microglia, ROS are mainly produced by the multi-subunit enzyme NADPH oxidase (NOX). Another reactive molecule, nitric oxide (NO), is produced by nitric oxide synthase (NOS) in microglia [70]. Whereas acute inflammation can be neuroprotective, it is the chronic inflammation state that is linked to neurodegenerative disease. Chronic inflammation will add to the load of ROS and potential DNA damage that can target neurons in the substantia nigra.

## 7. DNA Repair Deficiencies and Neurodegeneration

In the context of DNA damage in neurons, it is also of interest that several mouse models and human patients with DNA repair deficiencies, in particular with defects in transcription-coupled repair, show phenotypes of neurodegeneration [72,73,74,75]. Neurodegeneration occurs in these mice without deliberate exposures, suggesting that a form of endogenous DNA damage may trigger these events. These neurodegenerative mouse models include deficiencies in global nucleotide excision repair (NER), most prominently xeroderma pigmentosum group A (XPA) and ERCC1 [73,76], and defects in the transcription-coupled nucleotide excision repair genes Cockayne syndrome A and B (CSA and CSB) (Figure 2B) [77]. In transcription-coupled NER, lesions that stall RNA polymerase when present on the transcribed DNA strand promote the rapid recruitment of the NER complex, resulting in preferential repair of the transcribed relative to the non-transcribed DNA strand (Figure 2B) [78]. XPC knockout mice, which are proficient in transcription-coupled repair but lack global genome repair, do not show neurodegeneration, and human XP-C patients have only mild neuronal symptoms [73,79]. These data suggests that DNA damage in transcribed regions of the genome is important in neurodegeneration. Furthermore, mice deficient in genes of the base excision repair pathway (Figure 2B) also show neurodegeneration. The genes with this phenotype include *OGG1*, an enzyme which removes 8-oxoG from DNA and *MTH1*, which hydrolyzes 8-oxodGTP found in the damaged nucleotide pool [80,81]. The histone deacetylase HDAC1 modulates OGG1-initated 8-oxoG repair in the brain, highlighting an important interplay between epigenetic and genetic factors in the control of brain aging and neurodegenerative diseases [82]. Interestingly, the gene encoding MUTYH promotes neurodegeneration [80]. MUTYH is a repair enzyme that operates on oxidative DNA damage by excising mis-incorporated adenine bases that are found opposite to 8-oxoguanine but leaves 8-oxoG itself unrepaired. The results from these mouse models suggested indirectly but compellingly that 8-oxoG causes neurodegeneration [80]. Whether this mechanism is relevant for human PD is still unknown.

## 8. Potential Mechanisms of How DNA Damage or Repair Deficiency May Contribute to Parkinson’s Disease

Certain chemicals and several inherited mutations that all promote mitochondrial defects have clearly been linked to PD in animal models and in human pedigrees and now also in a large human epidemiological study [10]. However, the mechanisms regarding how these compounds and gene mutations cause the disease are unknown.

A common feature in mitochondrial dysfunction is the production of reactive oxygen species which may lead to DNA damage. One hypothesis is that ROS produced by dysfunctional mitochondria will lead to the formation of oxidative DNA damage in nuclear DNA. The unknown outcome is how such genome damage may lead to neurodegenerative diseases.

**Figure 4:**
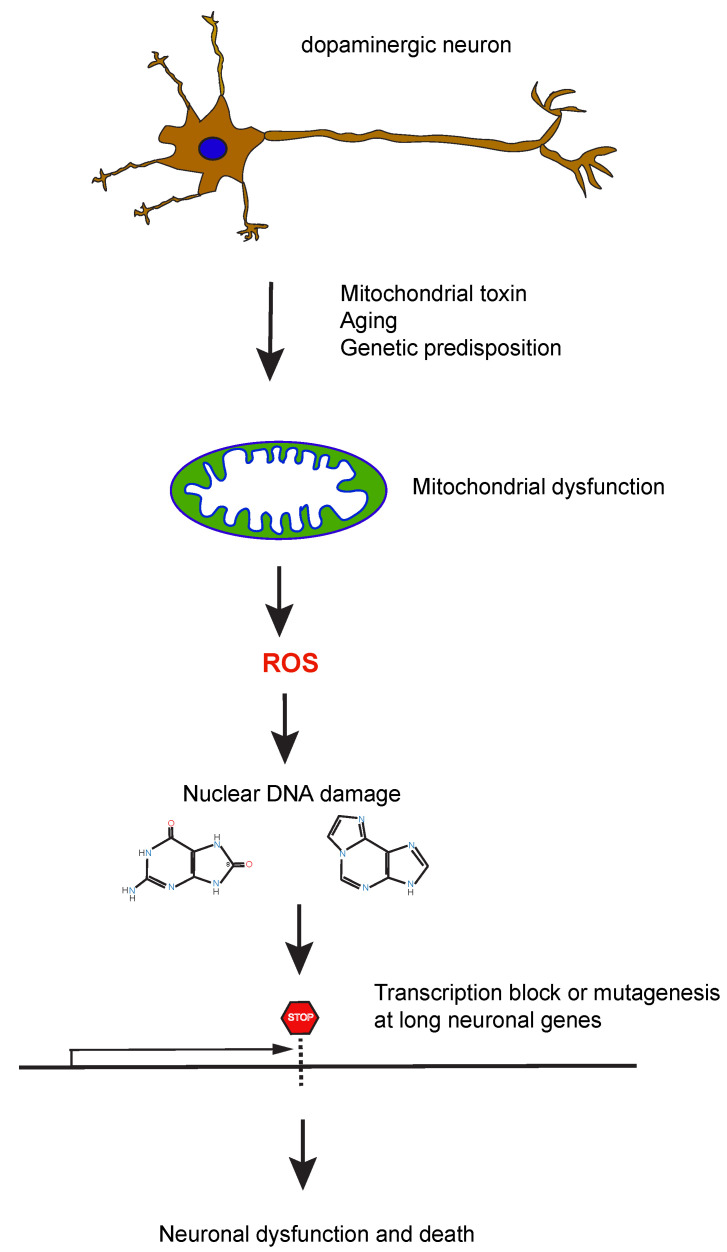
Hypothesis of how ROS generate detrimental oxidative DNA damage in long neuron-specific genes. DNA-damaging ROS are produced in dysfunctional mitochondria after exposure to mitochondrial toxins, during the aging process, or because of a genetic predisposition. ROS damages nuclear DNA, leading to the formation of transcription blocking lesions in long genes. The lesions may also cause permanent mutations leading to neuronal dysfunction and cell death.

It could be proposed that ROS-induced DNA damage or lack of its repair, when occurring in long neuron-specific genes [75,83], will lead to a reduction in transcript levels with the consequence of neuronal dysfunction, loss of neuronal identity, and dopaminergic cell death (Figure 4). Importantly, many genes that encode proteins with neuron-specific function are extraordinary long and often GC-rich [84,85,86,87], ranging in size from several hundred kilobases to well over a megabase [88]. The transcriptome of neurons is biased for having longer transcripts relative to other brain cells and relative to other tissues [83].

These long genes (>300 kilobases) often encode proteins involved in axon and synapse formation and neuronal cell adhesion and are often mutated in human neurodevelopmental disorders. Examples of such long and potentially disease-relevant genes are *NRXN3* (1700 kb) [89], *OXR1* (486 kb) [90], *RIT2* (376 kb) [91], *DLG2* (2177 kb) [92], *LSAMP* (647 kb) [93], *RBFOX1* (1698 kb) [94], *VPS13B* (868 kb) [95], *WWOX* (1117 kb) [96], *NFIA* (389 kb) [97], *SOX5* (1036 kb) [98], and *PARKIN* itself (1384 kb) [47]. Table 3 summarizes these genes and a few additional genes with very long transcription units. Genes with non-neuronal functions rarely have this exceptional length. Axonal degeneration appears to be an early neurodegenerative event in PD, and dopaminergic and excitatory synapses are substantially reduced in PD [99]. I predict that such long genes will contain large numbers of damaged DNA bases, predominantly in the form of 8-oxoguanine (8-oxoG), which is a major DNA damage product produced by ROS. This base lesion stalls or slows down RNA polymerase II [100,101]. Stalling is even more pronounced by the further oxidation products of 8-oxoG, spiroiminodihydantoin (Sp) and 5-guanidinohydantoin (Gh) as well as by DNA single-strand break repair intermediates, cyclopurines and exocyclic etheno base adducts produced by lipid peroxidation-derived aldehydes. Transcription blockage by DNA damage in long genes may lead to neurological dysfunction and death [75].

The general DNA damage level in long genes is likely further exacerbated by physiological DNA base turnover at the many intronic enhancer regions of such long neuronal genes, which is a process that produces oxidized 5-methylcytosines subject to base excision repair [102,103]. Neurons are notorious for having high levels of 5-methylcytosine oxidation [104], which is strongly enhanced in gene bodies of neuron-specific genes during neuronal differentiation [105]. It was indeed reported that very long genes show more frequently reduced expression during aging [106,107,108] and in Alzheimer’s disease brain [109]. In their study, Soheili-Nezhad et al. tried to connect this phenomenon to increased mutations developing with age [109]; however, mutations are probably too rare to explain a strong reduction in gene expression. Another recent study concluded that there is a gene length-associated transcriptome imbalance with age in humans and that it preferentially leads to a relative fold decrease in longer transcripts with the strongest effect in brain tissue [110]. Endogenous DNA damage has also been implicated to yield a gene length-associated decrease of the longest transcripts in a progeroid, DNA repair-deficient mouse model of aging [111]. When the DNA damage is converted to mutations, perhaps during erroneous repair events, mutations in protein-coding genes may permanently alter protein function in long-lived neurons, leading to haploinsufficiency or a complete loss of function when both copies of a gene are affected.

In addition to the loss of midbrain dopamine producing neurons, PD is characterized by the aggregation of alpha-synuclein into Lewy bodies and Lewy neurites, which are the major neuropathological hallmarks of the disease. As discussed extensively here, one other important hallmark of PD is mitochondrial dysfunction [5,112]. Alpha-synuclein aggregation and Lewy body formation also cause mitochondrial damage and dysfunction, although the mechanisms are not entirely understood [46,113]. Going both ways, perhaps in a vicious cycle, it has been shown that mitochondrial dysfunction and oxidative stress in turn cause alpha-synuclein aggregation [114].

## 9. Conclusions

In summary, this review highlights the emerging connections between mitochondrial dysfunction, a hallmark of PD, the formation of excess reactive oxygen species in dopaminergic neurons, both because of the mitochondrial defects and as an inherent property of the redox-active dopamine molecule, and the ensuing DNA damage. This DNA damage when occurring in the nuclear genome will be particularly detrimental for the expression of long neuron-specific genes and may cause the mutagenesis of long genes. A reduction in their expression due to this damage will lead to a loss of neuronal functions and eventually will result in the demise of dopaminergic neurons. Future studies will be needed to assess each step of the model and its validity as a whole.

## Figures and Tables

**Table 1:** Environmental toxins linked to Parkinson’s disease.

Chemical	Use	Mode of Action	Structure
MPTP	Synthetic chemical	Converted to neurotoxic MPP+ in the brain. Inhibits mitochondrial complex 1.	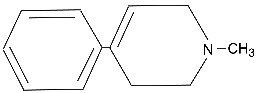
6-hydroxydopamine	Synthetic chemical used to induce Parkinsonism	Undergoes oxidation to quinones. Production of ROS.	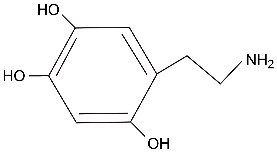
Trichloroethylene	Cleaning and degreasing agent	Inhibits mitochondrial complex 1. Carcinogenic.	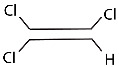
Rotenone	Insecticide, piscicide, and pesticide	Inhibits mitochondrial complex 1.	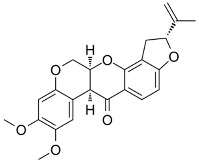
Paraquat	Herbicide	Production of ROS. Mitochondrial toxicity.	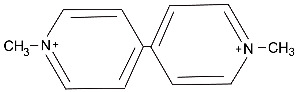

**Table 2:** Familial PD genes with a functional role in mitochondrial homeostasis.

Gene	Mutation Type	Mode of Action	Effect on Mitochondria	Reference
*SNCA*	Missense, amplification	Unknown. Disordered protein prone to aggregation	Deposition of aggregates inhibits mitochondrial function and produces ROS.	[46]
*PRKN*	Missense, copy number change, LOF	Ubiquitin ligase	Promotes mitochondrial quality control.	[47]
*PINK1*	Missense, deletion, LOF	Serin/threonine protein kinase	Recruits PRKN to mitochondria. Controls respiratory chain function.	[47]
*DJ-1*	Missense, LOF	Located at outer mitochondrial membrane.	Promotes mitochondrial function. Inhibits ROS formation.	[48]
*LRRK2*	Missense, GOF	Kinase and GTPase	Promotes mitophagy.	[49]
*VPS35*	Missense, D620N, GOF (?)	Vacuolar protein sorting	Loss of VPS35 causes mitochondrial dysfunction and fragmentation.	[50]
*ATP13A2*	Missense, LOF	Lysosomal protein	Loss of ATP13A2 increases ROS and cell death.	[51]
*VPS13C*	Missense, LOF	Vacuolar protein sorting	Role in normal mitochondrial biogenesis and function.	[52]
*CHCHD2*	Missense, T61I	CHCH domain containing protein	Maintains mitochondrial matrix structure.	[53]
*FBXO7*	Missense, LOF	Adapter protein for ubiquitin E3 ligase	Recruits PRKN to damaged mitochondria.	[54]
*PLA2G6*	Missense, LOF	Phospholipase	Maintains mitochondrial function.	[55]

**Table 3:** Examples of long genes with functions in axon and synapse formation, neuronal cell adhesion, or PD.

Gene	Name	Length	Presumed Function	Human Disorders
*PRKN*	Parkin	1379 kb	Ubiquitin ligase, regulates mitochondrial quality control	Familial PD gene
*NRXN3*	Neurexin 3	1695 kb	Cell adhesion molecule	Autism
*OXR1*	Oxidation resistance 1	484 kb	Critical for oxidative stress resistance of neurons	Cerebellar hypoplasia
*RIT2*	RIC-like protein	375 kb	Small GTPase Rit2 loss is causal for SNc cell death and motor dysfunction in mice	PD risk allele
*DLG2*	Disks large homolog 2	2169 kb	Synaptic protein, membrane-associated guanylate kinase	Neurodevelop-mental disorders
*LSAMP*	Limbic system associated membrane protein	644 kb	Cell adhesion molecule on axonal membranes	unknown
*RBFOX1*	RNA binding FOX1 homologue	1692	RNA binding protein involved in splicing	Neurodevelop-mental disorders
*VPS13B*	Vacuolar protein sorting-associated 13B	865 kb	Golgi associated protein	Autism, Cohen syndrome
*WWOX*	WW domain containing oxidoreductase	1112 kb	Multifunctional protein	Spinocerebellar ataxia, epileptic encephalopathy
*CNTNAP2*	Contactin-associated protein-like 2	2299 kb	Cell adhesion molecule	autism
*DAB1*	Disabled 1	1255 kb	Reelin signaling, critical for neurodevelopment	Neurodevelop-mental disorders
*SOX5*	SRY-related box 5	1033 kb	Transcription factor	Neurodevelop-mental disorder

## Data Availability

Not applicable.

## References

[1] Cherian A., Divya K.P., Vijayaraghavan A. (2023). Parkinson’s disease—Genetic cause. Curr. Opin. Neurol..

[2] Nalls M.A., Blauwendraat C., Vallerga C.L., Heilbron K., Bandres-Ciga S., Chang D., Tan M., Kia D.A., Noyce A.J., Xue A. (2019). Identification of novel risk loci, causal insights, and heritable risk for Parkinson’s disease: A meta-analysis of genome-wide association studies. Lancet Neurol..

[3] Lopez-Otin C., Blasco M.A., Partridge L., Serrano M., Kroemer G. (2023). Hallmarks of aging: An expanding universe. Cell.

[4] Antony P.M., Diederich N.J., Kruger R., Balling R. (2013). The hallmarks of Parkinson’s disease. FEBS J..

[5] Wilson D.M., Cookson M.R., Van Den Bosch L., Zetterberg H., Holtzman D.M., Dewachter I. (2023). Hallmarks of neurodegenerative diseases. Cell.

[6] Pavlou M.A.S., Outeiro T.F. (2017). Epigenetics in Parkinson’s Disease. Adv. Exp. Med. Biol..

[7] van Heesbeen H.J., Smidt M.P. (2019). Entanglement of genetics and epigenetics in Parkinson’s disease. Front. Neurosci..

[8] Ben-Shlomo Y., Darweesh S., Llibre-Guerra J., Marras C., San Luciano M., Tanner C. (2024). The epidemiology of Parkinson’s disease. Lancet.

[9] Ball N., Teo W.P., Chandra S., Chapman J. (2019). Parkinson’s Disease and the environment. Front. Neurol..

[10] Goldman S.M., Weaver F.M., Stroupe K.T., Cao L., Gonzalez B., Colletta K., Brown E.G., Tanner C.M. (2023). Risk of Parkinson disease among service members at marine corps base Camp Lejeune. JAMA Neurol..

[11] Wadman M. (2023). Solvent exposure strongly linked to Parkinson’s. Science.

[12] Dorsey E.R., Zafar M., Lettenberger S.E., Pawlik M.E., Kinel D., Frissen M., Schneider R.B., Kieburtz K., Tanner C.M., De Miranda B.R. (2023). Trichloroethylene: An invisible cause of Parkinson’s disease?. J. Parkinson’s Dis..

[13] De Miranda B.R., Greenamyre J.T. (2020). Trichloroethylene, a ubiquitous environmental contaminant in the risk for Parkinson’s disease. Environ. Sci. Process. Impacts.

[14] Liu M., Choi D.Y., Hunter R.L., Pandya J.D., Cass W.A., Sullivan P.G., Kim H.C., Gash D.M., Bing G. (2010). Trichloroethylene induces dopaminergic neurodegeneration in Fisher 344 rats. J. Neurochem..

[15] De Miranda B.R., Castro S.L., Rocha E.M., Bodle C.R., Johnson K.E., Greenamyre J.T. (2021). The industrial solvent trichloroethylene induces LRRK2 kinase activity and dopaminergic neurodegeneration in a rat model of Parkinson’s disease. Neurobiol. Dis..

[16] Liu M., Shin E.J., Dang D.K., Jin C.H., Lee P.H., Jeong J.H., Park S.J., Kim Y.S., Xing B., Xin T. (2018). Trichloroethylene and Parkinson’s disease: Risk assessment. Mol. Neurobiol..

[17] Martinez T.N., Greenamyre J.T. (2012). Toxin models of mitochondrial dysfunction in Parkinson’s disease. Antioxid. Redox Signal..

[18] Gash D.M., Rutland K., Hudson N.L., Sullivan P.G., Bing G., Cass W.A., Pandya J.D., Liu M., Choi D.Y., Hunter R.L. (2008). Trichloroethylene: Parkinsonism and complex 1 mitochondrial neurotoxicity. Ann. Neurol..

[19] Ibarra-Gutierrez M.T., Serrano-Garcia N., Orozco-Ibarra M. (2023). Rotenone-induced model of Parkinson’s disease: Beyond mitochondrial complex I inhibition. Mol. Neurobiol..

[20] Paul K.C., Krolewski R.C., Lucumi Moreno E., Blank J., Holton K.M., Ahfeldt T., Furlong M., Yu Y., Cockburn M., Thompson L.K. (2023). A pesticide and iPSC dopaminergic neuron screen identifies and classifies Parkinson-relevant pesticides. Nat. Commun..

[21] Bhatti J.S., Bhatti G.K., Reddy P.H. (2017). Mitochondrial dysfunction and oxidative stress in metabolic disorders—A step towards mitochondria based therapeutic strategies. Biochim. Biophys. Acta Mol. Basis Dis..

[22] Misrani A., Tabassum S., Huo Q., Tabassum S., Jiang J., Ahmed A., Chen X., Zhou J., Zhang J., Liu S. (2021). Mitochondrial Deficits With Neural and Social Damage in Early-Stage Alzheimer’s Disease Model Mice. Front. Aging Neurosci..

[23] Caboni P., Sherer T.B., Zhang N., Taylor G., Na H.M., Greenamyre J.T., Casida J.E. (2004). Rotenone, deguelin, their metabolites, and the rat model of Parkinson’s disease. Chem. Res. Toxicol..

[24] Tanner C.M., Kamel F., Ross G.W., Hoppin J.A., Goldman S.M., Korell M., Marras C., Bhudhikanok G.S., Kasten M., Chade A.R. (2011). Rotenone, paraquat, and Parkinson’s disease. Environ. Health Perspect.

[25] Gao H.M., Liu B., Hong J.S. (2003). Critical role for microglial NADPH oxidase in rotenone-induced degeneration of dopaminergic neurons. J. Neurosci..

[26] Barbeau A., Roy M., Bernier G., Campanella G., Paris S. (1987). Ecogenetics of Parkinson’s disease: Prevalence and environmental aspects in rural areas. Can. J. Neurol. Sci..

[27] Tangamornsuksan W., Lohitnavy O., Sruamsiri R., Chaiyakunapruk N., Norman Scholfield C., Reisfeld B., Lohitnavy M. (2019). Paraquat exposure and Parkinson’s disease: A systematic review and meta-analysis. Arch. Environ. Occup. Health.

[28] Paul K.C., Cockburn M., Gong Y., Bronstein J., Ritz B. (2024). Agricultural paraquat dichloride use and Parkinson’s disease in California’s Central Valley. Int. J. Epidemiol..

[29] Sharma P., Mittal P. (2024). Paraquat (herbicide) as a cause of Parkinson’s Disease. Parkinsonism. Relat. Disord..

[30] Chinta S.J., Woods G., Demaria M., Rane A., Zou Y., McQuade A., Rajagopalan S., Limbad C., Madden D.T., Campisi J. (2018). Cellular senescence is Induced by the environmental neurotoxin paraquat and contributes to neuropathology linked to Parkinson’s disease. Cell Rep..

[31] Cristovao A.C., Campos F.L., Je G., Esteves M., Guhathakurta S., Yang L., Beal M.F., Fonseca B.M., Salgado A.J., Queiroz J. (2020). Characterization of a Parkinson’s disease rat model using an upgraded paraquat exposure paradigm. Eur. J. Neurosci..

[32] Burns R.S., Chiueh C.C., Markey S.P., Ebert M.H., Jacobowitz D.M., Kopin I.J. (1983). A primate model of parkinsonism: Selective destruction of dopaminergic neurons in the pars compacta of the substantia nigra by N-methyl-4-phenyl-1,2,3,6-tetrahydropyridine. Proc. Natl. Acad. Sci. USA.

[33] Markey S.P., Johannessen J.N., Chiueh C.C., Burns R.S., Herkenham M.A. (1984). Intraneuronal generation of a pyridinium metabolite may cause drug-induced parkinsonism. Nature.

[34] Langston J.W., Ballard P., Tetrud J.W., Irwin I. (1983). Chronic Parkinsonism in humans due to a product of meperidine-analog synthesis. Science.

[35] Pantic I., Cumic J., Skodric S.R., Dugalic S., Brodski C. (2021). Oxidopamine and oxidative stress: Recent advances in experimental physiology and pharmacology. Chem. Biol. Interact..

[36] Lu X., Kim-Han J.S., Harmon S., Sakiyama-Elbert S.E., O’Malley K.L. (2014). The Parkinsonian mimetic, 6-OHDA, impairs axonal transport in dopaminergic axons. Mol. Neurodegener..

[37] Glinka Y., Tipton K.F., Youdim M.B. (1996). Nature of inhibition of mitochondrial respiratory complex I by 6-Hydroxydopamine. J. Neurochem..

[38] Dolle C., Flones I., Nido G.S., Miletic H., Osuagwu N., Kristoffersen S., Lilleng P.K., Larsen J.P., Tysnes O.B., Haugarvoll K. (2016). Defective mitochondrial DNA homeostasis in the substantia nigra in Parkinson disease. Nat. Commun..

[39] Nissanka N., Moraes C.T. (2018). Mitochondrial DNA damage and reactive oxygen species in neurodegenerative disease. FEBS Lett..

[40] Sian-Hülsmann J., Mandel S., Youdim M.B., Riederer P. (2011). The relevance of iron in the pathogenesis of Parkinson’s disease. J. Neurochem..

[41] Pyatha S., Kim H., Lee D., Kim K. (2022). Association between heavy metal exposure and Parkinson’s disease: A review of the mechanisms related to oxidative stress. Antioxidants.

[42] Trempe J.F., Gehring K. (2023). Structural mechanisms of mitochondrial quality control mediated by PINK1 and Parkin. J. Mol. Biol..

[43] Kumaran R., Cookson M.R. (2015). Pathways to Parkinsonism Redux: Convergent pathobiological mechanisms in genetics of Parkinson’s disease. Hum. Mol. Genet..

[44] Billingsley K.J., Barbosa I.A., Bandres-Ciga S., Quinn J.P., Bubb V.J., Deshpande C., Botia J.A., Reynolds R.H., Zhang D., Simpson M.A. (2019). Mitochondria function associated genes contribute to Parkinson’s Disease risk and later age at onset. NPJ Parkinsons Dis..

[45] Sun M.E., Zheng Q. (2023). The tale of DJ-1 (PARK7): A Swiss army knife in biomedical and psychological research. Int. J. Mol. Sci..

[46] Sohrabi T., Mirzaei-Behbahani B., Zadali R., Pirhaghi M., Morozova-Roche L.A., Meratan A.A. (2023). Common mechanisms underlying alpha-synuclein-induced mitochondrial dysfunction in Parkinson’s disease. J. Mol. Biol..

[47] Pereira S.L., Grossmann D., Delcambre S., Hermann A., Grunewald A. (2023). Novel insights into Parkin-mediated mitochondrial dysfunction and neuroinflammation in Parkinson’s disease. Curr. Opin. Neurobiol..

[48] Skou L.D., Johansen S.K., Okarmus J., Meyer M. (2024). Pathogenesis of DJ-1/PARK7-mediated Parkinson’s disease. Cells.

[49] Singh F., Ganley I.G. (2021). Parkinson’s disease and mitophagy: An emerging role for LRRK2. Biochem. Soc. Trans..

[50] Williams E.T., Chen X., Otero P.A., Moore D.J. (2022). Understanding the contributions of VPS35 and the retromer in neurodegenerative disease. Neurobiol. Dis..

[51] Dang T., Cao W.J., Zhao R., Lu M., Hu G., Qiao C. (2022). ATP13A2 protects dopaminergic neurons in Parkinson’s disease: From biology to pathology. J. Biomed. Res..

[52] Schreglmann S.R., Houlden H. (2016). VPS13C-another hint at mitochondrial dysfunction in familial Parkinson’s disease. Mov. Disord..

[53] Ren Y.L., Jiang Z., Wang J.Y., He Q., Li S.X., Gu X.J., Qi Y.R., Zhang M., Yang W.J., Cao B. (2024). Loss of CHCHD2 stability coordinates with C1QBP/CHCHD2/CHCHD10 complex impairment to mediate PD-linked mitochondrial dysfunction. Mol. Neurobiol..

[54] Burchell V.S., Nelson D.E., Sanchez-Martinez A., Delgado-Camprubi M., Ivatt R.M., Pogson J.H., Randle S.J., Wray S., Lewis P.A., Houlden H. (2013). The Parkinson’s disease-linked proteins Fbxo7 and Parkin interact to mediate mitophagy. Nat. Neurosci..

[55] Liu J., Tan J., Tang B., Guo J. (2024). Unveiling the role of iPLA(2)beta in neurodegeneration: From molecular mechanisms to advanced therapies. Pharmacol. Res..

[56] Kim H.W., Choi W.S., Sorscher N., Park H.J., Tronche F., Palmiter R.D., Xia Z. (2015). Genetic reduction of mitochondrial complex I function does not lead to loss of dopamine neurons in vivo. Neurobiol. Aging.

[57] Subrahmanian N., LaVoie M.J. (2021). Is there a special relationship between complex I activity and nigral neuronal loss in Parkinson’s disease? A critical reappraisal. Brain Res..

[58] Abascal F., Harvey L.M.R., Mitchell E., Lawson A.R.J., Lensing S.V., Ellis P., Russell A.J.C., Alcantara R.E., Baez-Ortega A., Wang Y. (2021). Somatic mutation landscapes at single-molecule resolution. Nature.

[59] Blokzijl F., de Ligt J., Jager M., Sasselli V., Roerink S., Sasaki N., Huch M., Boymans S., Kuijk E., Prins P. (2016). Tissue-specific mutation accumulation in human adult stem cells during life. Nature.

[60] Müller-Nedebock A.C., Brennan R.R., Venter M., Pienaar I.S., van der Westhuizen F.H., Elson J.L., Ross O.A., Bardien S. (2019). The unresolved role of mitochondrial DNA in Parkinson’s disease: An overview of published studies, their limitations, and future prospects. Neurochem. Int..

[61] Müller W.E., Eckert A., Kurz C., Eckert G.P., Leuner K. (2010). Mitochondrial dysfunction: Common final pathway in brain aging and Alzheimer’s disease--therapeutic aspects. Mol. Neurobiol..

[62] Parker W.D., Parks J.K., Swerdlow R.H. (2008). Complex I deficiency in Parkinson’s disease frontal cortex. Brain Res..

[63] Breitzig M., Bhimineni C., Lockey R., Kolliputi N. (2016). 4-Hydroxy-2-nonenal: A critical target in oxidative stress?. Am. J. Physiol. Cell Physiol..

[64] Alam Z.I., Jenner A., Daniel S.E., Lees A.J., Cairns N., Marsden C.D., Jenner P., Halliwell B. (1997). Oxidative DNA damage in the parkinsonian brain: An apparent selective increase in 8-hydroxyguanine levels in substantia nigra. J. Neurochem..

[65] Jin S.G., Meng Y., Johnson J., Szabo P.E., Pfeifer G.P. (2022). Concordance of hydrogen peroxide-induced 8-oxo-guanine patterns with two cancer mutation signatures of upper GI tract tumors. Sci. Adv..

[66] Puspita L., Chung S.Y., Shim J.W. (2017). Oxidative stress and cellular pathologies in Parkinson’s disease. Mol. Brain.

[67] Guo J.D., Zhao X., Li Y., Li G.R., Liu X.L. (2018). Damage to dopaminergic neurons by oxidative stress in Parkinson’s disease (Review). Int. J. Mol. Med..

[68] Miller M.B., Huang A.Y., Kim J., Zhou Z., Kirkham S.L., Maury E.A., Ziegenfuss J.S., Reed H.C., Neil J.E., Rento L. (2022). Somatic genomic changes in single Alzheimer’s disease neurons. Nature.

[69] Harding O., Holzer E., Riley J.F., Martens S., Holzbaur E.L.F. (2023). Damaged mitochondria recruit the effector NEMO to activate NF-kappaB signaling. Mol. Cell.

[70] Pajares M., Rojo A.I., Manda G., Boscá L., Cuadrado A. (2020). Inflammation in Parkinson’s disease: Mechanisms and therapeutic implications. Cells.

[71] Bido S., Muggeo S., Massimino L., Marzi M.J., Giannelli S.G., Melacini E., Nannoni M., Gambare D., Bellini E., Ordazzo G. (2021). Microglia-specific overexpression of alpha-synuclein leads to severe dopaminergic neurodegeneration by phagocytic exhaustion and oxidative toxicity. Nat. Commun..

[72] Wang H., Lautrup S., Caponio D., Zhang J., Fang E.F. (2021). DNA damage-induced neurodegeneration in accelerated ageing and Alzheimer’s disease. Int. J. Mol. Sci..

[73] Jaarsma D., van der Pluijm I., de Waard M.C., Haasdijk E.D., Brandt R., Vermeij M., Rijksen Y., Maas A., van Steeg H., Hoeijmakers J.H. (2011). Age-related neuronal degeneration: Complementary roles of nucleotide excision repair and transcription-coupled repair in preventing neuropathology. PLoS Genet..

[74] Jeppesen D.K., Bohr V.A., Stevnsner T. (2011). DNA repair deficiency in neurodegeneration. Prog. Neurobiol..

[75] Kajitani G.S., Nascimento L.L.S., Neves M.R.C., Leandro G.D.S., Garcia C.C.M., Menck C.F.M. (2021). Transcription blockage by DNA damage in nucleotide excision repair-related neurological dysfunctions. Semin. Cell Dev. Biol..

[76] Sepe S., Milanese C., Gabriels S., Derks K.W., Payan-Gomez C., van IJcken W.F., Rijksen Y.M., Nigg A.L., Moreno S., Cerri S. (2016). Inefficient DNA repair Is an aging-related modifier of Parkinson’s disease. Cell Rep..

[77] Weidenheim K.M., Dickson D.W., Rapin I. (2009). Neuropathology of Cockayne syndrome: Evidence for impaired development, premature aging, and neurodegeneration. Mech. Ageing Dev..

[78] Selby C.P., Lindsey-Boltz L.A., Li W., Sancar A. (2023). Molecular mechanisms of transcription-coupled repair. Annu. Rev. Biochem..

[79] Fang E.F., Scheibye-Knudsen M., Brace L.E., Kassahun H., SenGupta T., Nilsen H., Mitchell J.R., Croteau D.L., Bohr V.A. (2014). Defective mitophagy in XPA via PARP-1 hyperactivation and NAD(+)/SIRT1 reduction. Cell.

[80] Sheng Z., Oka S., Tsuchimoto D., Abolhassani N., Nomaru H., Sakumi K., Yamada H., Nakabeppu Y. (2012). 8-Oxoguanine causes neurodegeneration during MUTYH-mediated DNA base excision repair. J. Clin. Investig..

[81] Cardozo-Pelaez F., Sanchez-Contreras M., Nevin A.B. (2012). Ogg1 null mice exhibit age-associated loss of the nigrostriatal pathway and increased sensitivity to MPTP. Neurochem. Int..

[82] Pao P.C., Patnaik D., Watson L.A., Gao F., Pan L., Wang J., Adaikkan C., Penney J., Cam H.P., Huang W.C. (2020). HDAC1 modulates OGG1-initiated oxidative DNA damage repair in the aging brain and Alzheimer’s disease. Nat. Commun..

[83] Zylka M.J., Simon J.M., Philpot B.D. (2015). Gene length matters in neurons. Neuron.

[84] Raychaudhuri S., Korn J.M., McCarroll S.A., International Schizophrenia C., Altshuler D., Sklar P., Purcell S., Daly M.J. (2010). Accurately assessing the risk of schizophrenia conferred by rare copy-number variation affecting genes with brain function. PLoS Genet..

[85] Smith D.I., Zhu Y., McAvoy S., Kuhn R. (2006). Common fragile sites, extremely large genes, neural development and cancer. Cancer Lett..

[86] King I.F., Yandava C.N., Mabb A.M., Hsiao J.S., Huang H.S., Pearson B.L., Calabrese J.M., Starmer J., Parker J.S., Magnuson T. (2013). Topoisomerases facilitate transcription of long genes linked to autism. Nature.

[87] Gabel H.W., Kinde B., Stroud H., Gilbert C.S., Harmin D.A., Kastan N.R., Hemberg M., Ebert D.H., Greenberg M.E. (2015). Disruption of DNA-methylation-dependent long gene repression in Rett syndrome. Nature.

[88] Wei P.C., Chang A.N., Kao J., Du Z., Meyers R.M., Alt F.W., Schwer B. (2016). Long neural genes harbor recurrent DNA break clusters in neural stem/progenitor cells. Cell.

[89] Zheng J.J., Li W.X., Liu J.Q., Guo Y.C., Wang Q., Li G.H., Dai S.X., Huang J.F. (2018). Low expression of aging-related NRXN3 is associated with Alzheimer disease: A systematic review and meta-analysis. Medicine.

[90] Oliver P.L., Finelli M.J., Edwards B., Bitoun E., Butts D.L., Becker E.B., Cheeseman M.T., Davies B., Davies K.E. (2011). Oxr1 is essential for protection against oxidative stress-induced neurodegeneration. PLoS Genet..

[91] Kearney P.J., Zhang Y., Tan Y., Kahuno E., Conklin T.L., Fagan R.R., Pavchinskiy R.G., Shafer S.A., Yue Z., Melikian H.E. (2023). Rit2 silencing in dopamine neurons drives a progressive Parkinsonian phenotype. bioRxiv.

[92] Griesius S., O’Donnell C., Waldron S., Thomas K.L., Dwyer D.M., Wilkinson L.S., Hall J., Robinson E.S.J., Mellor J.R. (2022). Reduced expression of the psychiatric risk gene DLG2 (PSD93) impairs hippocampal synaptic integration and plasticity. Neuropsychopharmacology.

[93] Jagomae T., Singh K., Philips M.A., Jayaram M., Seppa K., Tekko T., Gilbert S.F., Vasar E., Lillevali K. (2021). Alternative promoter use governs the expression of IgLON cell adhesion molecules in histogenetic fields of the embryonic mouse brain. Int. J. Mol. Sci..

[94] Prashad S., Gopal P.P. (2021). RNA-binding proteins in neurological development and disease. RNA Biol..

[95] Montillot C., Skutunova E., Ayushma, Dubied M., Lahmar A., Nguyen S., Peerally B., Prin F., Duffourd Y., Thauvin-Robinet C. (2023). Characterization of Vps13b-mutant mice reveals neuroanatomical and behavioral phenotypes with females less affected. Neurobiol. Dis..

[96] Aldaz C.M., Hussain T. (2020). WWOX loss of function in neurodevelopmental and neurodegenerative disorders. Int. J. Mol. Sci..

[97] Sagner A., Zhang I., Watson T., Lazaro J., Melchionda M., Briscoe J. (2021). A shared transcriptional code orchestrates temporal patterning of the central nervous system. PLoS Biol..

[98] Li L., Medina-Menendez C., Garcia-Corzo L., Cordoba-Beldad C.M., Quiroga A.C., Calleja Barca E., Zinchuk V., Munoz-Lopez S., Rodriguez-Martin P., Ciorraga M. (2022). SoxD genes are required for adult neural stem cell activation. Cell Rep..

[99] Gcwensa N.Z., Russell D.L., Cowell R.M., Volpicelli-Daley L.A. (2021). Molecular mechanisms underlying synaptic and axon degeneration in Parkinson’s Ddsease. Front. Cell. Neurosci..

[100] Kuraoka I., Endou M., Yamaguchi Y., Wada T., Handa H., Tanaka K. (2003). Effects of endogenous DNA base lesions on transcription elongation by mammalian RNA polymerase II. Implications for transcription-coupled DNA repair and transcriptional mutagenesis. J. Biol. Chem..

[101] Tornaletti S., Maeda L.S., Kolodner R.D., Hanawalt P.C. (2004). Effect of 8-oxoguanine on transcription elongation by T7 RNA polymerase and mammalian RNA polymerase II. DNA Repair.

[102] Caldecott K.W., Ward M.E., Nussenzweig A. (2022). The threat of programmed DNA damage to neuronal genome integrity and plasticity. Nat. Genet..

[103] Pfeifer G.P. (2021). DNA repair in neurons and its possible link to the epigenetic machinery at enhancers. Epigenomics.

[104] Jin S.G., Wu X., Li A.X., Pfeifer G.P. (2011). Genomic mapping of 5-hydroxymethylcytosine in the human brain. Nucleic Acids Res..

[105] Hahn M.A., Qiu R., Wu X., Li A.X., Zhang H., Wang J., Jui J., Jin S.G., Jiang Y., Pfeifer G.P. (2013). Dynamics of 5-hydroxymethylcytosine and chromatin marks in mammalian neurogenesis. Cell Rep..

[106] Gyenis A., Chang J., Demmers J., Bruens S.T., Barnhoorn S., Brandt R.M.C., Baar M.P., Raseta M., Derks K.W.J., Hoeijmakers J.H.J. (2023). Genome-wide RNA polymerase stalling shapes the transcriptome during aging. Nat. Genet..

[107] Papadakis A., Gyenis A., Pothof J., Hoeijmakers J.H.J., Papantonis A., Beyer A. (2023). Age-associated transcriptional stress due to accelerated elongation and increased stalling of RNAPII. Nat. Genet..

[108] Ibanez-Sole O., Barrio I., Izeta A. (2023). Age or lifestyle-induced accumulation of genotoxicity is associated with a length-dependent decrease in gene expression. iScience.

[109] Soheili-Nezhad S., van der Linden R.J., Olde Rikkert M., Sprooten E., Poelmans G. (2021). Long genes are more frequently affected by somatic mutations and show reduced expression in Alzheimer’s disease: Implications for disease etiology. Alzheimers Dement..

[110] Stoeger T., Grant R.A., McQuattie-Pimentel A.C., Anekalla K.R., Liu S.S., Tejedor-Navarro H., Singer B.D., Abdala-Valencia H., Schwake M., Tetreault M.P. (2022). Aging is associated with a systemic length-associated transcriptome imbalance. Nat. Aging.

[111] Vermeij W.P., Dolle M.E., Reiling E., Jaarsma D., Payan-Gomez C., Bombardieri C.R., Wu H., Roks A.J., Botter S.M., van der Eerden B.C. (2016). Restricted diet delays accelerated ageing and genomic stress in DNA-repair-deficient mice. Nature.

[112] Toomey C.E., Heywood W.E., Evans J.R., Lachica J., Pressey S.N., Foti S.C., Al Shahrani M., D’Sa K., Hargreaves I.P., Heales S. (2022). Mitochondrial dysfunction is a key pathological driver of early stage Parkinson’s. Acta Neuropathol. Commun..

[113] Mahul-Mellier A.L., Burtscher J., Maharjan N., Weerens L., Croisier M., Kuttler F., Leleu M., Knott G.W., Lashuel H.A. (2020). The process of Lewy body formation, rather than simply alpha-synuclein fibrillization, is one of the major drivers of neurodegeneration. Proc. Natl. Acad. Sci. USA.

[114] Li W., Fu Y., Halliday G.M., Sue C.M. (2021). PARK genes link mitochondrial dysfunction and alpha-synuclein pathology in sporadic Parkinson’s disease. Front. Cell. Dev. Biol..

